# Teachers’ Readiness to Implement Robotics in Education: Validation and Measurement Invariance of TRi-Robotics Scale via Confirmatory Factor Analysis and Network Psychometrics

**DOI:** 10.3390/bs15091227

**Published:** 2025-09-10

**Authors:** Theano Papagiannopoulou, Julie Vaiopoulou, Dimitrios Stamovlasis

**Affiliations:** 1 School of Philosophy and Education, Aristotle University of Thessaloniki, 54124 Thessaloniki, Greece; papagiat@edlit.auth.gr (T.P.); stadi@edlit.auth.gr (D.S.); 2 Department of Education, University of Nicosia, 2417 Nicosia, Cyprus; 3 School of Humanities, Hellenic Open University, 26335 Patras, Greece

**Keywords:** educational robotics, psychometric properties, network psychometrics, validation

## Abstract

The incorporation of educational robotics (ER) into classroom learning has emerged as a significant goal in contemporary education, with instructors assuming a pivotal role. Recent research has shown the influence of teachers’ perceptions of ER and their self-efficacy on the learning process, while the primary goal in these inquiries is to the development of appropriate scales that guarantee correct measurements. Serving this goal, the present study presents the TRi-Robotics scale and its psychometric properties, which assesses teachers’ readiness to integrate ER into their classrooms. TRi-Robotics is a novel multidimensional tool that integrates self-efficacy, commitment, and affective conditions, validated through both CFA and network psychometrics. The proposed 14-item scale is three-dimensional and includes self-efficacy (SE), commitment (C), and affective conditions (AC). The validation procedure included the customary Exploratory and Confirmatory Factor Analysis, applied to a sample of 817 in-service teachers. Reliability analysis showed satisfactory internal consistency, while measurement invariance for gender was sustained. Furthermore, network psychometrics was applied via Exploratory Graph Analysis (EGA), which supported the proposed structure and its dimensionality and measurement invariance. The TRi-Robotics scale proved a valid instrument with satisfactory psychometric properties, and it is a significant asset to implement in educational and psychological research for testing further research hypotheses.

## 1. Introduction

In the early 21st century, the fourth industrial revolution (4IR) achieved significant advancements, resulting in several technological innovations that educational institutions across are adopting. As artificial intelligence, robotics, and virtual reality advance (González-Pérez & Ramírez-Montoya, 2022), the significance of education aimed at enhancing creativity becomes more pronounced. Organizational leaders need to revise their visions, objectives, structures, and management practices to match with the challenges, demands, and opportunities arising from this transformation. Educational institutions need to adjust to the rapid advancements and changing skill demands of future graduates (Hyunjin & Tongjin, 2020; Savela et al., 2018; Singaram et al., 2023). The application of robotics in education fosters the personal and psychoemotional development of children (Fitter et al., 2020). At present, ER is being integrated into multiple facets of education, predominantly as a compulsory element of both formal and nonformal educational endeavors. It is employed in initiatives that assess many facets of educational robotics for students with special needs (Daniela & Lytras, 2019). It has the capacity to significantly impact student learning by providing a pragmatic and project-based framework that enables interdisciplinary exploration of disciplines such as science, technology, engineering, and mathematics (STEM) (Sapounidis et al., 2023).

Multiple studies have demonstrated that the integration of robots into teaching and learning enhances students’ enthusiasm and performance, as well as their professional outlook towards STEM subjects (Chiang et al., 2022; Ching et al., 2019). Furthermore, it serves as a tool to enhance students’ 21st century capabilities, including creative thinking, decision-making, problem-solving, information, and communication skills, and fosters their capacity to collaborate in teams (Ali et al., 2023; Darmawansah et al., 2023). Research has indicated that students who engaged in robot project-based activities achieved superior scores compared to those who did not receive training using this pedagogical approach (Leonard et al., 2016; Ouyang & Xu, 2024). Furthermore, robotics integrates other pedagogical approaches and ideas, such as project-based learning, real-world problem-solving, constructivism, and cooperation (Petraki & Herath, 2022; Zadok, 2019). In educational robotics activities, robots serve as manipulatives that offer rapid feedback. This allows children to get a deeper understanding of abstract ideas and problem-solving methods as learning gets more hands-on, tangible, and interactive (Ching & Hsu, 2024). The progress and application of robotics will have a profound impact on how people learn and participate in their work (Hrastinski et al., 2019). Therefore, modern educational institutions must provide organizations to equip learners with the necessary knowledge and abilities to succeed in future job markets that are yet to develop (World Bank, 2019).

Teachers have a significant impact on how learners perceive and respond to the ER. Consequently, educators play a crucial role in implementing ER in the classroom and integrating it into the curriculum (Schina et al., 2021a). To fully leverage the advantages of ER, it is essential to improve and assess their competence and skill in integrating ER into their teaching methodologies. Consequently, readiness is crucial, particularly for educators who must adapt to any novel and challenging framework. Educators must possess the requisite pedagogical and knowledge-based competencies to effectively transform informational content into educational formats (Petraki & Herath, 2022). These formats should also be adaptable to fit the diverse backgrounds and abilities of students. The use of passive instructive strategies can impede students’ understanding and hamper their ability to achieve learning goals (OECD, 2021). Nonetheless, it remains essential to delineate the specific facets of teachers’ professionalism that are most engaged and from which they obtain the most significant advantages for ER learning outcomes. It is essential to identify a validated instrument to assess the appropriate disposition of teachers and their adequate preparation. The outcomes of these measurements may facilitate the comparison and enhancement of the various training trajectories for educators in Greece. Numerous instruments exist for examining educators’ perspectives, perceptions, self-efficacy, and attitudes regarding ER (Di Battista et al., 2020; Negrini, 2020; Piedade, 2021; Reich-Stiebert & Eyssel, 2016; A. L. L. Tang et al., 2023), yet none have undergone validation. Among the limited validated studies (Dorotea et al., 2021; Jaipal-Jamani & Angeli, 2017; Tzagaraki et al., 2022) none takes into account teachers’ affective conditions. This gap is critical, as prior instruments primarily focused on cognitive or attitudinal dimensions without systematically addressing the emotional readiness of teachers (Piedade, 2021; Reich-Stiebert & Eyssel, 2016). By validating a multidimensional scale that integrates self-efficacy, commitment, and affective conditions, the present study introduces a novel and comprehensive framework for assessing teachers’ readiness to implement ER. Positive emotions enhance teachers’ dedication to their profession, resulting in increased energy and effort devoted to their work. This can directly and favorably impact teachers’ performance (Dilekçi et al., 2025). Moreover, prior studies indicate that elevated levels of educator self-efficacy are positively correlated with positive emotional states (e.g., happiness and pride) and negatively correlated with negative emotional states (e.g., anger and anxiety) (Burić et al., 2020). The objective of the study was to establish the validity of TRi-Robotics questioner, examine its psychometric features, and assess its applicability in the educational setting of Greece. The scale delineates three dimensions via exploratory factor analysis (EFA), confirmatory factor analysis (CFA), and EGA network analysis: i.e., commitment, affective conditions, and self-efficacy. The examination of these characteristics may influence the educational system and impact various elements, including decisions related to teacher training programs or teaching methodologies appropriate for diverse educational environments (Schina et al., 2021b). The questionnaire results may offer insights for institutional training agencies regarding educator training processes, as well as teachers’ interest in ER. This may also reflect teachers’ self-efficacy, which significantly impacts the initial years of teaching, as well as their collaborative abilities, crucial for facilitating education (Holmqvist & Lelinge, 2021). The responses from this questionnaire can offer a practical tool to guide targeted interventions and policy design. By measuring teachers’ pedagogical self-efficacy, commitment, and affective readiness, educational institutions can identify specific areas where support is needed. For example, policymakers might prioritize ongoing professional development, peer mentoring programs, or communities of practice that focus on long-term engagement rather than initial skill acquisition.

The Preceding Illustrates the Significance of Teacher Readiness, Elucidating the Increasing Interest in and Pursuit of a Robust Theoretical Framework to Address Challenging Inquiries Regarding the Implementation of Curriculum Modifications. Consequently, Research Has Undertaken This Work and Extensively Examined the Assumptions of Instructors’ Mindsets and Readiness to Implement ER. This Study Seeks to Develop and Present a Tool for Assessing Educators’ Readiness to Integrate Extensive Reading into Their Pedagogical Practices. The Device Will Yield Accurate Measurements of the Latent Variables Employed in This Evaluation. 

### 1.1. The Integration of Robotics into the Field of Education

Robotics is highly regarded as an effective approach for engaging kids in STEM disciplines, enabling them to explore, create, and apply knowledge to solve real-world problems (Ching et al., 2019). The use of educational robots corresponds with several contemporary learning theories, including active learning principles (Taylor et al., 2021), Papert’s constructionism (Giacomassi Luciano et al., 2019), and the social constructivism thesis (Martínez-Tenor et al., 2019). Constructionist learning entails self-directed inquiry, the creation of personally significant material or virtual products, and cooperation with peers and/or stakeholders. The creation of artifacts fosters creativity in their design and development, alongside critical thinking and problem-solving skills to learn from errors and enhance artifacts iteratively (Broza et al., 2023). Constructionism is an appropriate methodology to enhance computer science education for teachers who participate in coding initiatives, design, and execute pertinent projects, and collaborate with peers while utilizing technologies (e.g., robots and simulations) governed by a programming language (Vasconcelos et al., 2025). Principles of constructionism and constructivism suggest that students attain deeper understanding more efficiently when they actively participate in the construction of their own knowledge through the creation and interaction with a virtual or physical artifact, such as robotics, in a collaborative learning setting (Dorotea et al., 2021; Lee, 2016). From this perspective, it is posited that students might improve their learning efficacy by interacting with tangible objects within the framework of genuine, real-world activities and problems. This method entails a supervised and collaborative procedure that integrates peer contributions. During the construction, programming, or interaction with a robotic device, students have the opportunity to use critical and creative thinking while engaging in hands-on learning (Chalmers & Nason, 2017). The integration of robots in education positively influences student behavior and development, especially in problem-solving skills and collaboration (Damith Herath, 2022), motivation to learn (Borzovs et al., 2016; Deublein et al., 2018), and overall enjoyment and participation in the classroom (Narbutaitė et al., 2018). ER is becoming more prevalent in classrooms as a means of implementing activities that promote the growth of students’ computational thinking (CT) competencies and foster the acquisition of 21st century abilities (Ching & Hsu, 2024; Papadakis & Kalogiannakis, 2022). Such activities typically involve students practicing issue decomposition, abstraction, computational reasoning, debugging, repetition, and generalization, which are the six main aspects of computational thinking (Shute et al., 2017). Moreover, the observed benefits include the practical application of programming and STEM ideas (Jormanainen & Tukiainen, 2020). By constructing and coding robots, educators can effectively incorporate principles from technology, engineering, and computer programming into their teaching (Drakatos & Stavridis, 2023; Fegely & Tang, 2022). STEM education provides a platform to cultivate creativity, analytical thinking, communication, and collaboration, with robotics serving as an effective instrument that enhances experiential learning across both robotics and STEM disciplines (Torres & Inga, 2025). ER offers advantages such as flexibility, open-source development, and intuitive interactions, enabling students to investigate and address real-world challenges, thereby effectively engaging youth in STEM education (Shang et al., 2023). Employing STEM principles via robotics can improve kids’ understanding of math and science while also fostering their enthusiasm and cultivating ambitions regarding potential STEM studies (Arís & Orcos, 2019; Erol & Canbeldek Erol, 2023). Technology and educational robotics are integral to STEM education, enabling the incorporation of fundamental ideas like engineering principles and interdisciplinary methodologies (Darmawansah et al., 2023). The implementation of robots in the classroom promotes student engagement, aids in the understanding of intricate subjects, and boosts motivation for studying (Menekse et al., 2017), hence significantly influencing students’ academic performance (Witherspoon et al., 2016). Robots are generally perceived as an innovative technological instrument that fosters enthusiasm in classrooms, promotes student engagement, and cultivates a conducive learning environment (K. Wang et al., 2023).

Despite the availability of numerous tools, misunderstanding persists regarding the integration of robots in education and the assessment of the effects of these technological innovations. A lack of instructional support for educators may induce reluctance in their use of new technology in the classroom. The lack of explicit standards for integrating robotics into education and the difficulty in assessing the complex skills necessitated by these new practices are obstructing educators and institutions from fully leveraging and investigating the benefits that technology may provide (Malvezzi et al., 2021; Mury et al., 2022; Screpanti et al., 2021). Furthermore, the absence of generally available resources in educational environments and the inadequate training of educators to connect robotics with STEM disciplines are significant barriers to the widespread incorporation of ER into conventional classroom teaching (Schina et al., 2021a; Stokes et al., 2022).

The decisions and actions of educators in the classroom significantly influence teaching approaches and student educational outcomes (Y. Tang & Hu, 2022; Tzagaraki et al., 2022). When educators recognize a subject, such as robotics, as significantly valuable, they tend to offer more comprehensive and engaging learning experiences that foster advanced cognitive skills and the capacity to address complex issues, which are crucial for developing robotics literacy. The enthusiasm of a teacher for a specific subject might enhance their teaching efficacy (Shahmoradi et al., 2024). However, in addition to having positive attitudes, teachers must also have relevant subject knowledge and technological skills in order to efficiently instruct technology-related subjects (An et al., 2022). Instructors are more likely to hold an adverse perspective on using technology in their teaching practices when they possess a weak comprehension of that subject matter and lack good instructional strategies (Negrini, 2020).

The perspectives, attitudes, and technological proficiencies of educators are regarded as significant factors in the acceptance of technology. They must monitor the various tools available, select the most appropriate for their requirements, and design an activity that achieves the desired objective, whether it pertains to computational thinking, cooperation, communication, or mathematics. This task necessitates that educators augment their digital competencies and advance pedagogical and didactic innovation (Screpanti et al., 2021). Moreover, a significant correlation appears between educators’ views towards technology and their behaviors in educational environments (Kalogiannakis et al., 2018; Vidal-Hall et al., 2020). The self-efficacy beliefs and knowledge of education practitioners, coupled with uncertainty or apprehension about educational technology, can profoundly influence their confidence in developing digital educational experiences centered on STEM and CT (MacDonald et al., 2020). In this context, any novel medium can be effectively integrated and maintained, contingent upon educators’ readiness to incorporate technology in a student-centered manner; specifically, educators need to (a) be cognizant of emerging innovative technologies; (b) embrace innovative technologies that provide significant advantages; (c) possess assurance that technologies can be utilized safely and securely; (d) exhibit strong confidence in their capacity to employ the technology appropriately (Casey et al., 2021). Conversely, ER can assist instructors in enhancing their engagement with STEM topics, rendering computational thinking and STEM activities more attractive to both teachers and pupils. Teachers’ previous involvement in robotics education can prompt them to reevaluate their instructional strategies and incorporate interactive teaching methodologies, such as student-centered pedagogy, which, when implemented in a student-centered framework, facilitates students’ comprehension and evaluation of a technology’s potential (Çetin & Demircan, 2020; Papadakis et al., 2021), the present paper informs the literature with a valid instrument to assess educators’ readiness to implement ER in their teaching practice to achieve their educational goals.

### 1.2. Teachers’ Self-Efficacy in Their Knowledge and Teaching of ER

Teachers’ self-efficacy regarding technological innovations and their incorporation into the classroom has been a longstanding topic of study in the discipline of education. The cultivation of self-efficacy is a complex process shaped by numerous factors. Self-efficacy is a dynamic capacity that involves the coordination of cognitive, social, emotional, and behavioral capacities to achieve a desired objective and it is evidenced by the unwavering execution of a task, despite encountering obstacles (Bandura, 1977). Self-efficacy has an impact on the selection of activities, the establishment of goals, the level of persistence in pursuing a goal, and the amount of effort invested (Artino, 2012). Pedagogical content self-efficacy is widely recognized as a crucial attribute of a teacher and encompasses the teacher’s knowledge of the curricula and the instructional strategies, the development of lesson plans focused on achieving learning outcomes, confidence in teaching using educational technologies, and proficiency in assessing students (Canbazoğlu et al., 2013; Liepa, 2024).

A robust curriculum and adequate teacher training are essential prerequisites for the implementation of ER in education. Instruction of robotics inside the classroom should emphasize the cultivation of interdisciplinary cognitive skills, essential for achievement throughout the curriculum. These skills include collaboration, problem-solving, logical thinking, and creative design (An et al., 2022). Instructors find it challenging to incorporate coding, robotics, and computational thinking despite the abundance of resources, apps, tasks, and enthusiasm in robotics education. Research indicates that teachers lack the necessary understanding, particularly when it comes to robotics, to effectively integrate robotics into their classroom instruction (Benitti & Spolaôr, 2017). Self-assurance and knowledge are signs of an effective use of educational robots by instructors to enhance their utilization in students’ learning activities (Dorotea et al., 2021). Lack of confidence and low self-efficacy using robotics to teach may be correlated with a lack of experience applying robotics projects or content knowledge (Piedade, 2021); thus, it is imperative for teachers to acquire pedagogical and content knowledge.

### 1.3. Teachers’ Level of Readiness from an Affective Perspective

There is a growing interest in integrating technology, including ER, into the classroom because of the numerous advantages it offers to pupils. Nevertheless, studies regarding the impact of emerging technologies on teachers, which facilitate enhanced student learning, are limited.

The teaching profession is characterized by the existence of various role relationships, the growing external demand for accountability, and the intricacy of the task (Reich-Stiebert & Eyssel, 2016). The increased availability of educational robots, thanks to advancements in their software and hardware technologies, along with the introduction of new computer science and robotics topics in school curricula, such as computational thinking, has led to a greater willingness among schools to invest in educational robots (Alam, 2022). Nevertheless, teachers often experience a significant amount of emotional distress (Eddy et al., 2020) and might feel worried or require assistance in implementing robots in their classrooms (Shahmoradi et al., 2024). A previous study found that the perceived usefulness of robotics declined dramatically as teachers implemented it in their classrooms due to unforeseen obstacles (Chevalier et al., 2020). Educators must preconfigure the robots before the class, ensuring they are sufficiently charged. Moreover, unexpected technology malfunctions may lead to robot failures, potentially resulting in a loss of students’ time and requiring substantial alterations to teachers’ lesson plans (Shahmoradi et al., 2019). Additionally, teachers encounter challenges in tracking students’ progress due to the varied learning approaches utilized by pupils, leading to an uncertain educational environment (Shahmoradi et al., 2024).

Moreover, teachers’ distrust or anxiety about educational technology, along with their self-efficacy beliefs and expertise, can have a substantial influence on their confidence in designing digital educational experiences (Papadakis et al., 2021). Notwithstanding the evident enthusiasm of instructors for ER activities, a considerable proportion encounter ambiguity, concern, or even trepidation around the integration of technology into their regular instructional practices (Di Battista et al., 2023). Utilization of educational technologies necessitate alterations in educators’ pedagogical approaches or impose pressure to develop technological competencies, resulting in repercussions such as social and emotional well-being (Fernández-Batanero et al., 2021). Educators’ well-being, often measured by the frequency of experiencing feelings of happiness, may be a crucial aspect in facilitating learning and fostering creative thinking, essential in robotics (Smyrnova-Trybulska et al., 2024). The well-being of teachers impacts their motivation, teaching efficacy, and work satisfaction, thus influencing student results and the school environment. Research indicates that self-efficacy may affect a teacher’s resilience, allowing them to manage the stressors and responsibilities of the profession more effectively (Y. Wang & Pan, 2023). Teachers with elevated self-efficacy tend to view challenges as opportunities for growth and development instead than insurmountable obstacles. Ultimately, self-efficacy has been associated with total well-being, including the emotional, behavioral, and social dimensions of teachers’ lives (X. Wang et al., 2024).

### 1.4. Teachers’ Commitment

Teacher commitment refers to a teacher’s continuous passion, enthusiasm, and considerable involvement in their professional responsibilities and their school (Park, 2005). The degree of dedication that teachers exhibit regarding their organization and occupation has a substantial impact on the quality and efficacy of their instruction, particularly in the setting of school reforms that require instructors to assume several responsibilities (Yang et al., 2019). Committed teachers are more likely to actively engage in their profession, collaborate with their colleagues, and pursue professional development opportunities. Furthermore, they are more likely to foster strong relationships with pupils and provide a supportive learning environment (Yusof et al., 2021). Researchers have discovered that school support, including effective leadership from the principle, active teacher involvement, and opportunities for professional development, significantly contributes to enhancing teacher commitment (Pan, 2023). An inclusive and cooperative educational environment is linked to heightened teacher self-assurance, which is crucial for successful instruction and knowledge acquisition (Mansor et al., 2021). Teachers with a high level of confidence are more likely to use effective teaching methods and often demonstrate a high level of proficiency in incorporating technology into their instructional practices, leading to improved academic achievement among their students (Berg et al., 2024; Yanış & Yürük, 2021). Teachers’ motivation to use education robots was significantly predicted by their level of commitment to technology. Teachers with an increased interest in modern technology were more likely to utilize educational robots compared to teachers with less interest in technological matters (Reich-Stiebert & Eyssel, 2016). The instructor plays a crucial role in motivating students in their academic work and instilling positive mindsets, as the teacher wields significant control over how robotics is perceived by the students (Benitti & Spolaôr, 2017).

### 1.5. Objectives of Research and Research Hypotheses

The study assumes the obligation of investigating the conjectures related to teachers’ mindset and readiness for implementing ER. This work aims to contribute to the field by developing and providing a valid tool for assessing the readiness of educators incorporate ER in their teaching methods. This is an important presupposition for accurately evaluating the latent variables in question and further testing any related research hypothesis. Thus, the research questions in this endeavor are associated with the dimensionality of the TRi-Robotics scale, its construct validity, the measurement invariance across genders, and reliability issues. Moreover, this work has a methodological dimension, in which, via the application network psychometrics, further support of the findings and additional information are pursued. To date, no prior study in ER education has employed network psychometrics to validate a readiness instrument. Thus, the TRi-Robotics scale not only fills a conceptual gap by integrating affective readiness, but also introduces methodological innovation that strengthens construct validity.

## 2. Materials and Methods

### 2.1. Participants and Procedures

The data were collected through an online survey distributed to in-service educators across various educational levels and specialties. A total of 817 participants completed the questionnaire in its entirety, after being contacted through the school email. Prior to analysis, the dataset was reviewed for inconsistencies or invalid responses. Notably, there was no missing data, as the online platform was configured to require responses to all items before submission. Table 1 displays the descriptive statistics summary. The final sample comprised 817 educators, with 536 employed in elementary schools and 281 in secondary schools. A predominant 78.9% of participants were female, with ages spanning from 46 to 65 years (mean = 46.96, SD = 9.338). A total of 49.2% possessed teaching experience between 14 and 26 years (mean = 18.2, SD = 9.870). Furthermore, 57.8% of the participants possessed a master’s degree, 38.2% had engaged in ER training programs, and 26.1% had integrated a relevant program into their classroom. The self-administered questionnaire was submitted via an online form. Educators were contacted via email to convey the research’s objectives, safety protocols, and participation options, allowing for anonymous responses at their convenience. The procedure is considered as opportunity sampling, with data collection at each stage according to the regulations established by the Ethics and Deontology Committee.

### 2.2. Instrument

The construction of the TRi-Robotics scale was based on theoretical underpinnings of similar works (Papagiannopoulou et al., 2023). The development of the TRi-Robotics scale was attained from an extended pool of items via an exploratory procedure involving factor analyses and theoretical considerations. The final form of this scale was proposed as a three-dimensional scale with 14 items including the following dimensions: self-efficacy (5 items), affective conditions (4 items), and commitment (5 items). In the data collection procedure, a 5-point Likert scale was used, marked from 1 (does not describe me at all) to 5 (absolutely describes me). The questionnaire also includes inquiries regarding demographics and personal traits, among which, gender was used to test the measurement invariance.

### 2.3. Analysis I: Exploratory and Confirmatory Factor Analysis

The first psychometric approach follows the common procedure of Exploratory Factor Analysis (EFA) applied to one half of the sample, while the other part was used for confirmatory procedures. In the EFA, Principal Axis Factoring (PAF) was used with promax rotation and parallel analysis to examine the factor structure of the psychological construct corresponding to teachers’ readiness for implementing educational robotics. Subsequently, Confirmatory Factor Analysis (CFA) was applied to the other half of the data, supporting, ergo, the validity of the scale. The measurement model’s goodness-of-fit for the three-dimensional structure, which was theoretically interpretable, was assessed via multiple fit indices, such as Chi-squared, CFI, TLI, and RMSEA. In addition, reliability measures were calculated using Cronbach’s alpha and McDonald’s omega coefficients for the measurement’s internal consistency.

Furthermore, a measurement invariance analysis was conducted to assess if the scale structure was the same across gender and teaching experience. The measurement invariance was carried out in four steps. It started with the *configural invariance* test which is the least restrictive model and the baseline. Then, the *metric invariance* concerned the factor loadings in the groups, followed by the *scalar invariance* test that examined whether the item intercepts were equivalent and ends with the *strict invariance* model (Chen, 2007). The comparison between measurement invariance models was carried out via the *χ*^2^ difference test, along with the other indices, on which the suggestive values are ΔCFI < 0.01, ΔTLI < 0.01 and ΔRMSEA < 0.015, for rejecting the null hypothesis that corresponds to invariance (Cheung & Rensvold, 2002; Hair et al., 2014).

### 2.4. Analysis II: Network Psychometrics

Network analysis has been introduced in psychological science to overcome the limitations of psychometric methods originating from the assumption of latent variables and the known principle of independence (Borsboom & Cramer, 2013; Fried & Cramer, 2017). Thus, besides traditional factor analysis methods, network psychometrics based on network analysis (Golino & Epskamp, 2017), which is based on different theoretical assumptions, can be implemented. Contrary to the traditional view that presupposes the existence of a latent entity as the common cause for observables, the network method considers that the co-occurrence of the latter (human behaviors) ascends from mutual influence or associations among these observed variables (Christensen & Golino, 2021).

In network psychometrics, variables are conceptualized as nodes of a network, while the edges or connections between them correspond to their associations. The clustering of these nodes allows the identification of latent groups as communities of variables without the need to explicitly assume the presence of a latent construct in the traditional view. Mathematically, it usually engages computation of partial correlations using a Gaussian Graphical Model with GLASSO regularization and the Extended Bayesian Information Criterion (EBIC) algorithm for model selection (Golino & Epskamp, 2017). Exploratory Graph Analysis (EGA) employs the Walktrap community detection algorithm (Pons & Latapy, 2006), which utilizes random walks methods to determine the number and structure of the communities within a network. It has been shown that EGA can be more efficient than parallel analysis and provide more accurate estimates (Golino & Epskamp, 2017), even in cases of unidimensional structures (Christensen et al., 2023; Golino et al., 2020). EGA is extensively used in psychometric network research in conjunction with the common factor analysis for establishing firm results. Moreover, centrality indices can be calculated, such as betweenness, closeness, strength, and expected influence, to evaluate the importance of each node (Borsboom & Cramer, 2013). The information regarding the significance has theoretical merit and can also be useful in practical applications and specifically to targeted interventions aiming at changing the attitudinal or cognitive variables. In addition, the analyses include measures of the stability of the networks and the accuracy of edge-weights and centrality, which can be assessed via bootstrapped methods (Epskamp et al., 2018).

## 3. Results

### 3.1. Exploratory Factor Analysis

The Kaiser–Meyer–Olkin index (0.960) and Bartlett’s test of sphericity (*Chi-square* = 11,678.68, *df* = 91, *p* = 0.000) suggested sufficient variance for factor analysis. The Exploratory Factor Analysis (EFA) using Principal Axis Factoring (PAF) with oblique promax rotation and parallel analysis provided the structure depicted in Table 1. The three dimensions explained 76.3% of the variance (14 items), while each of factors’ commitment (RCOM, 5 items), self-efficacy (RSE, 5 items) and affective conditions (RAC, 4 items) explained 29.8% (eigenvalue 4.17), 27.4% (eigenvalue 3.83), and 19.1% (eigenvalue 2.68), respectively. The factor loadings were >0.55. Table 2 displays the factors’ loadings and the uniqueness, or the variance that is not shared with other variables, as well as the three-dimensional structure.

Using Cronbach’s alpha and McDonald’s omega, the four factors’ reliability measures were calculated as follows: RCOM (*α* = 0.922/*ω* = 0.921), RSE (*α* = 0.936/*ω* = 0.937), and RAC (*α* = 0.937/*ω* = 0.943). These reliability indices indicate that the internal consistency of the current measurements is adequate.

Table 3 shows the correlation matrix of the three dimensions, means and standard deviations, and the reliability indices. Commitment correlates with self-efficacy (*r* = 0.670, *p* < 0.001), affective conditions (*r* = 0.835, *p* < 0.001), and affective conditions with self-efficacy (*r* = 0.772, *p* < 0.001).

### 3.2. Confirmatory Factor Analysis

CFA showed that the three-dimensional model fitted the actual data satisfactorily [*χ*^2^ (74) = 235.076, *p* < 0.001, *TLI* = 0.965, *CFI* = 0.972, *GFI* = 0.977, *NNFI* = 0.965, *RMSEA* = 0.073 (0.063–0.084), and *SRMR* = 0.036]. Table 4 displays the CFA measuring model. 

The measurement invariance test for gender is presented in Table 5. The Chi-square difference (Δ*χ*^2^) and the differences in CFI and TLI were used to conclude for each of the models. Comparing the *configural invariance model* with the restrictive *metric invariance* model, the difference is statistically insignificant *p*-value (*p* = 0.90) and thus the meaning of the construct is the same for males and females, and the factor variances and covariances are likewise. For the next tests (Table 4), however, the differences are statistically significant, denoting rejection of the measurement invariance hypothesis. Nevertheless, examining the differences of the other fit indices [ΔCFI < 0.01, ΔTLI < 0.01, and ΔRMSEA < 0.015] it can be concluded that even though there is a lack of invariance for some parameters, the overall model fit is not practically affected regarding the two genders (Vandenberg & Lance, 2000). The limitations here are that the two groups are not numerically equal, so the findings are cautiously reported.

### 3.3. Network Psychometrics

The network analysis was based on the 14 items of the TRi-Robotics scale by applying Exploratory Graph Analysis (EGA) using R software (version 4.0.0, available at https://cran.r-project.org/) and the R-package “qgraph” for network estimation (Epskamp et al., 2012). The nodes of the network were empirical indices or responses to questionnaire items, and the edges were partial correlations between each pair of nodes after controlling for all the other nodes. Our data were considered as ordinal in nature, and the Gaussian Graphical Model was estimated using the *glasso* algorithm (graphical Least Absolute Shrinkage and Selection Operator) in combination with Extended Bayesian Information Criteria (EBIC) (Tibshirani, 1996). The tuning parameter set as 0.5 (Foygel & Drton, 2010; Isvoranu et al., 2017) and the arrangement of the nodes in the network was achieved by the force-directed Fruchterman–Reingold algorithm (Fruchterman & Reingold, 1991).

**Figure 1:**
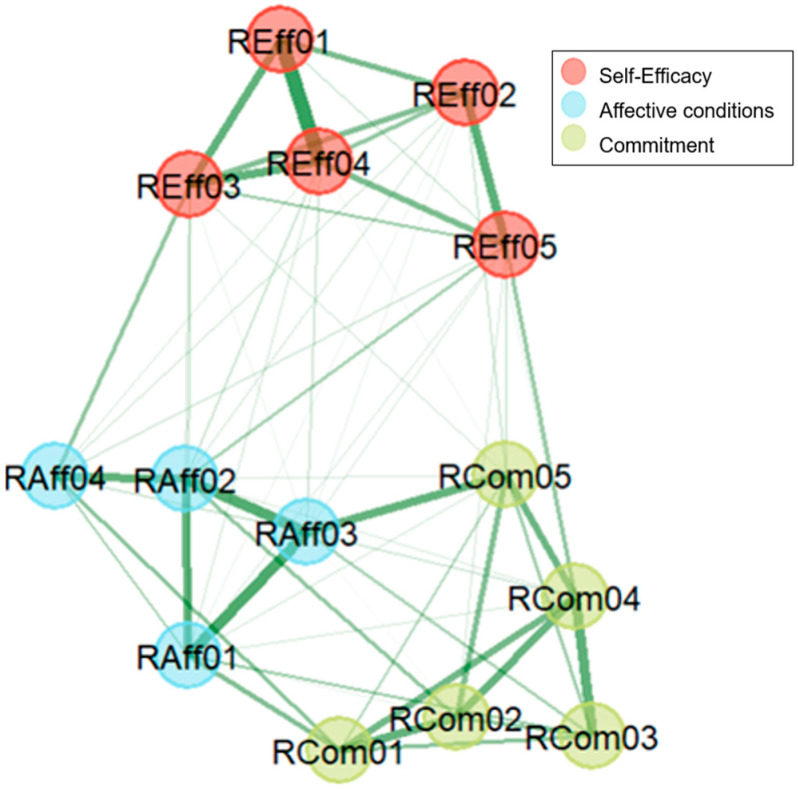
Exploratory Graph Analysis: the dimensional structure of the TRi-Robotics scale. The detected communities represent the three dimensions, i.e., self-efficacy, affective conditions, and commitment. Stronger connections are represented with thicker lines.

By applying the walktrap algorithm, we identified three communities (Figure 1) which were consistent with the theoretical factor model proposed in the TRi-Robotics scale. The 14 items were parcellated into the (1) “self-efficacy” factor, (2) “affective conditions” factor, and (3) “commitment” factor.

In addition, using calculated centrality indices, we evaluated the significance of the nodes. The centrality measures were betweenness, closeness strength, and expected influence (Borsboom & Cramer, 2013). A node (item) with high betweenness implies that it engages in the shortest routes between pairs of the other nodes. High closeness indicates close connections with all the other nodes. Strength represents the sum of the absolute weights of all the edges connected to the node. Expected influence is the strength accounting for the negative edges that might exist (Robinaugh et al., 2016).

**Figure 2:**
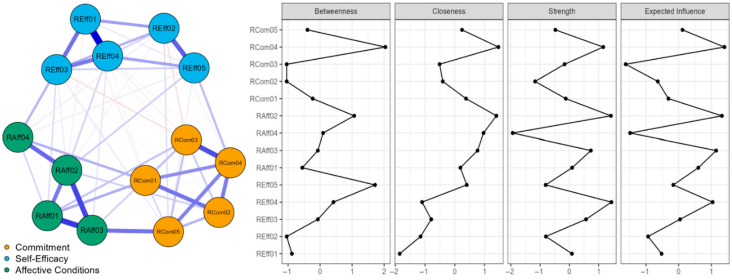
TRi-Robotics item network. The blue edges indicate positive partial correlations and edges in red indicates negative partial correlations. Stronger connections are represented with thicker lines. Centrality measures in Z-scores of betweenness, closeness strength, and expected influence.

From Figure 2, it can be observed that in community “commitment” the node/item RCom04 (I am willing to seek more information from professionals regarding educational robotics applications to enhance my students’ learning) has the greatest betweenness, closeness strength, and expected influence. In community “affective conditions” the node/item RAff02 (I am excited about the implementation of educational robotics activities in the classroom) has the greatest betweenness, closeness strength, and expected influence. For community “self-efficacy” the node REff05 (I could assess learning outcomes in robotics learning activities) is the most important item, having the greatest values in all centrality measures.

The stability of the network and the accuracy of edge-weights and centrality were assessed via a bootstrapped approach (Epskamp et al., 2018). The accuracy of edge-weights was estimated by calculated non-parametric bootstrapped Confidence Intervals (CIs) using 2.500 permutations and depicted in the corresponding diagram. The diagrams that concern this measure are provided in the Supplementary Materials, Figure S1. Narrow bootstrapped CIs indicate low sampling variability in edge-weights and an accurate network. The stability of strength was evaluated using case-dropping subset bootstrap to judge how well the order of centralities was retained in portions merely of data. The correlation stability coefficient (CSC) was implemented for measuring the maximum drop in proportions to retain a correlation of 0.70 in at least 95% of the sample (Epskamp et al., 2018). Stability analysis for the estimated network in the whole sample showed relatively narrow bootstrapped CIs, suggesting reliable edge-weights. All the diagrams that concern the accuracy and stability, measures are provided in the Supplementary Materials, Figures S1–S3.

**Figure 3:**
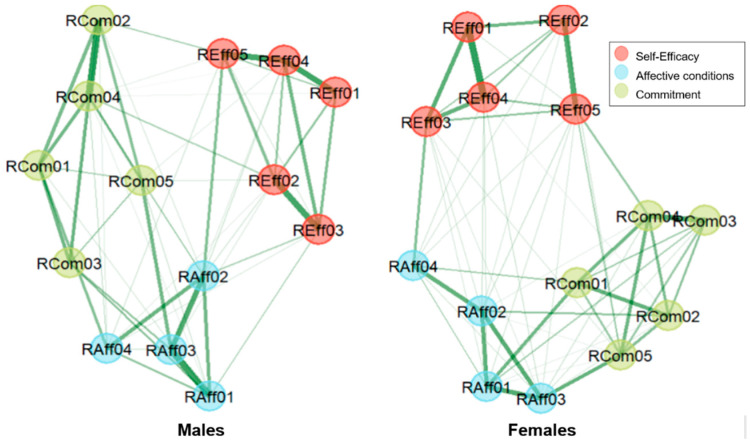
The network structure of TRi-Robotics scale for males and females. The detected communities represent the three dimensions, i.e., self-efficacy, affective conditions, and commitment. Stronger connections are represented with thicker lines.

To evaluate the measurement invariance between genders, we used the Network Comparison Test. Having estimated the corresponding networks for males and females (Figure 3), we examined the invariance of these network structures, global strength, and edge-weights by implementing two-tailed permutation tests (van Borkulo et al., 2023). The test is based on the maximum difference between matrices containing all connections. The global strength of a network, that is defined as the sum of the absolute edge-weights of all pairs of nodes in the network, was calculated and comparisons were made for the two networks (males and females). The algorithm also includes the false discovery rate (FDR) correction for addressing multiple comparisons of edge-weights, and adjusted *p*-values (Benjamini & Hochberg, 1995). The significance threshold for the invariance test of network structure and global strength, and for differences on edge-weights, was set at *p*-value < 0.05. The Network Comparison Test was also performed in R statistical software.

Figure 3 shows the network structures of the TRi-Robotics scale for males (A) and females (B), clearly demonstrating the three dimensions, self-efficacy, affective conditions, and commitment. Tests for the accuracy and stability measures for the two networks were also performed and showed satisfactory results. The corresponding diagrams are provided in the Supplementary Materials (Figures S4–S9).

**Figure 4:**
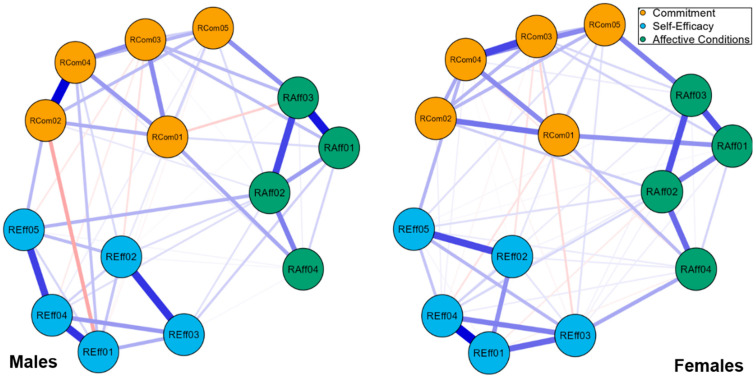
TRi-Robotics item network for males and females. The blue edges indicate positive partial correlations and edges in red indicate negative partial correlations. Stronger connections are represented with thicker lines.

Performing the Network Comparison Test, no significant differences were found across male and female groups, indicative of the invariance of network structure (M = 0.289, *p* = 0.060) and global strength (S = 0.271, *p* = 0.406) (Figure 4). Thus, the networks of items/observables in TRi-Robotics scale are not significantly different between males and females.

In addition, using calculated centrality measures, one could observe some detailed differences between the two networks, which, even if the whole structures are equivalent, might have a special interest (Figure 4). For example, the commitment expressed by RCom04 item has greater betweenness centrality for females, while the opposite holds in RCom02 item, and the affective conditions expressed by RAff04 has greater betweenness centrality for females also. These kinds of differences need further search and elaboration and might be meaningful and useful in gender differences studies.

## 4. Discussion

### 4.1. Conclusions

This paper presents the development and validation of the TRi-Robotics scale, an instrument measuring teacher readiness to implement robotics in education, via two methodological approaches, the traditional and the network psychometrics, in order to enhance the validity of the findings. Although EFA and CFA provide a comprehensive analytical framework, other approaches, such as EGA, yield further insights into the fundamental structure of robotics readiness questioner.

The proposed three-dimensional model was based on theoretical underpinnings that were elaborated in the introduction section, which are consistent with the relevant psychological processes involved. The model was demonstrated via traditional factor analyses, while reliability analysis showed satisfactory internal consistency. The TRi-Robotics scale includes three factors, namely pedagogical self-efficacy, robotics commitment, and affective conditions. These dimensions, which are correlated, offer valuable insights into the conceptualization of educational robotics use, and the theoretical framework is fully supported by educational psychology premises.

Through the present study, the interrelated factors of self-efficacy, commitment, and instructors’ affective conditions can be further examined in relation to the integration of educational robotics (ER) into daily teaching practices. Educators need to be sufficiently prepared and supported to effectively serve as change agents. This requires both essential content knowledge and pedagogical skills to engage students in critical discussions on sustainability and motivate them to take action. Self-efficacy is a vital motivational element in education, affecting teachers’ enthusiasm, commitment, and instructional methods (Daumiller et al., 2025). Moreover, teachers’ emotional health is a crucial subject of investigation because of its substantial influence on their professional efficacy and the overall standard of education. The well-being of teachers impacts their motivation, teaching efficacy, commitment, and job satisfaction, subsequently influencing student outcomes and the school environment (Burić & Macuka, 2018; X. Wang et al., 2024). Given the beneficial influence of self-efficacy and commitment, policymakers must implement and showcase programs that enhance teacher well-being and classroom engagement (Shu, 2022).

The literature documenting findings that demonstrate robust correlations between instructors’ characteristics, the adoption of emerging technologies, and subsequent learning outcomes is consistently expanding (e.g., Ali et al., 2023; Zeng et al., 2022). In addition to accumulating facts, research and theory development necessitate the utilization of sophisticated methodological techniques, which, in turn, demand accurate measurements. All the studies, with notable insights presented in the introduction concerning educators’ attitudes and behaviors towards the utilization of robotics, represent a dynamic research domain, wherein the advancement of theoretical frameworks is heavily reliant on precise and credible measurements; consequently, the TRi-Robotics scale will be an essential instrument for ensuring the validity and reliability of assumptions.

The novel part of the present work is its methodological dimension; that is, via network analysis, it provided further support on the psychometric properties of TRi-Robotics scale. Previous validation studies in related fields (e.g., Di Battista et al., 2023; Reich-Stiebert & Eyssel, 2016) relied exclusively on traditional approaches such as EFA and CFA. In contrast, the application of network psychometrics in the present study offers an innovative analytical perspective, enabling the identification of central items and structural stability that cannot be captured by conventional methods. This dual-method validation enhances the robustness of the TRi-Robotics scale and distinguishes it from prior work. The proposed three-dimensional structure that includes self-efficacy, commitment, and affective conditions, was also established by network psychometrics, along with the measurement invariance across genders. The application of network psychometrics in validating the TRi-Robotics scale presents an innovative analytical perspective that enhances conventional techniques. CFA evaluates predicted latent structures and validates the adequacy of a specified model, whereas network analysis instead illustrates direct relationships among the observed variables (items) as nodes interconnected by edges, facilitating a more empirical investigation of the scale’s architecture. This methodology elucidates the interactions of items within and between dimensions (e.g., affective states, self-efficacy, and commitment) and can identify clusters of items (communities) or central nodes that exert significant influence within the network. Summarizing, network representations could identify clustering among nodes that correspond to observable variables and separate them into distinct dimensions, in line with the hypothesized theoretical constructs. The strong associations among them were also measured via centrality indices, and the three-dimensional structure was evaluated in terms of its stability and accuracy via bootstrap methods. Additional information provided is the crucial role of certain nodes, described in terms of betweenness, closeness, strength, and expected influence. These centrality measures have theoretical and practical implications. Certain nodes, for example with high degree or other centrality measures, are the most important in the network of conceptual structures. This knowledge, besides a potential theoretical value, informs and becomes useful to practice, and specifically to the targeted interventions that are directed to modify or change participants’ attitudes or behavior on the issue under study.

There is a growing interest in the development of a solid theoretical framework to tackle complex questions about the implementation of curriculum changes. Consequently, this study thoroughly analyzed the assumptions of teachers’ self-efficacy and their preparedness to adopt ER. The analysis of these features may affect the educational system and influence many factors, including judgments on teacher training programs or teaching approaches suitable for distinct educational contexts. This may also indicate instructors’ self-efficacy, which profoundly influences the early years of teaching, along with their collaboration skills, essential for promoting education. By assessing instructors’ pedagogical self-efficacy, commitment, and emotional preparedness, educational institutions can pinpoint precise areas requiring support. Policymakers may support continuous professional development peer mentorship initiatives, or communities of practice that emphasize sustained participation over initial skill acquisition. Ultimately, the degree of instructors’ readiness for ER can be precisely evaluated using the TRi-Robotics scale, an instrument substantiated by two psychometric methodologies. To our knowledge, this is the first validated instrument in ER education that simultaneously captures self-efficacy, commitment, and affective conditions, while also employing both CFA and network psychometrics. The novelty of the present endeavor ensures that the TRi-Robotics scale provides not only reliable measurement but also unique insights into the interrelationships among readiness dimensions, marking a significant advancement in the field. The validation of Tri-Robotics with two robust methodologies reinforces the foundational theoretical framework and demonstrates the efficacy of the suggested questionnaire as a reliable instrument, which will be an essential resource for future efforts to test further hypotheses in educational and psychological research about the implementation of educational robotics.

Concerning diverse educational contexts, the adoption of the TRI-Robotics scale offers both significant advantages and challenges, particularly when considering disparities in technological infrastructure and resources. On one hand, the tool provides a valid framework for assessing teachers’ readiness to integrate robotics, serving as a valuable resource for needs assessment and targeted professional development planning. It is especially useful in identifying non-technical barriers, such as low self-efficacy or affective resistance, which may exist even in well-equipped environments. Furthermore, the scale can help inform policy decisions by highlighting readiness gaps due to beliefs, attitudes, and institutional support structures.

On the other hand, its implementation in low-resource or developing settings may face challenges. Limited access to robotics equipment, outdated infrastructure, and budgetary constraints may lead to context-bound responses, where teachers evaluate their readiness hypothetically rather than based on real experience.

### 4.2. Limitations and Areas for Future Investigation

This study, while significant and beneficial, possesses certain limitations. The demographic distribution is skewed towards older age groups, requiring caution when generalizing findings to younger cohorts. Moreover, dependence exclusively on self-report instruments poses the possibility of response biases and social desirability influences. Concerning network methodologies, they provide a rich historical background and numerous prospective applications within the field of psychology. Nonetheless, there are significant yet underappreciated constraints in psychometric network studies of variables assessing symptoms, beliefs, or qualities.

This study is one of the preliminary efforts to fulfill the criteria for accurate research measurements. Consequently, more validation is necessary by the incorporation of more samples and duplicated experiments. The constraints stem from the utilization of cross-sectional data obtained using self-reported instruments and the restricted nature of the sample. Scenario-based assessments may be utilized in conjunction with teacher-report questionnaires to assess educators’ attitudes toward practices in ER.

Furthermore, to attain a more profound understanding of the elements affecting educators’ self-efficacy and competence in teaching ER or implementing an ER curriculum, it is recommended to do extensive qualitative research, including instructor interviews and classroom observations. To improve cross-cultural comparability and foster acceptability, future research must assess the reliability of the scale across various cultural contexts. Additional cross-cultural replications are essential, and the measurement invariance could be broadened to include other individual differences.

Moreover, it is essential to recognize that the proposed factorial structure of the scale should not be regarded as definitive. Rather, it possesses the capacity for expansion to incorporate more aspects informed by diverse theoretical premises. This expansion would improve the depiction of the fundamental characteristic of readiness. Finally, supplementary validation techniques may be instituted in the future, including machine learning and artificial intelligence methodologies that are now gaining prominence.

To enhance the efficacy of the TRi-Robotics scale, additional research is required that examines various sample compositions and sizes, and it should also be adapted and validated in additional languages. Moreover, regarding technological constraints and training deficiencies, it is advisable that subsequent applications of the TRi-Robotics scale incorporate control parameters to contextualize responses. Furthermore, longitudinal validation studies could investigate the evolution of self-efficacy and affective attitudes prior to and following structured robotics training. Incorporating the scale into professional growth programs and gathering pre/post data would improve its predictive validity and ensure alignment between measurement outcomes and actual classroom implementation.

## Figures and Tables

**Table 1:** Descriptive statistics.

Variable	Category	Count (N)	Total %
Gender	Male	172	21.1%
	Female	645	78.9%
Age	25–35	110	13.5%
	36–46	247	30.3%
	47–65	458	56.2%
Teaching Specialization	1. Greek Language	43	5.3%
	2. Mathematics	27	3.3%
	3. Physics	43	5.3%
	4. English Language	36	4.4%
	5. Computer Science	107	13.1%
	6. Physical Education	22	2.7%
	7. Kindergarten Teachers	200	24.5%
	8. Primary School Teachers	231	28.3%
	9. Other Specialties	108	13.2%
Teaching Experience (Years)	1–13	248	30.4%
	14–26	402	49.3%
	27–40	166	20.3%
Implementation of Robotics Program	Yes	213	26.1%
	No	604	73.9%
Participation in Robotics Seminar	Yes	312	38.2%
	No	505	61.8%

**Table 2:** Factor loadings of three-dimensional structure.

	RCOM *α* = 0.922 *ω* = 0.921	RSE *α* = 0.936 *ω* = 0.937	RAC *α* = 0.937 *ω* = 0.943	Uniqueness
**RCom04**—I am willing to seek more information from professionals regarding educational robotics applications to enhance my students’ learning.	0.929			0.166
**RCom03**—I am willing to attend educational robotics courses to enhance my knowledge.	0.855			0.313
**RCom02**—I would devote time to seek effective strategies prior to integrating educational robotics activities into my courses.	0.821			0.272
**RCom01**—I would devote the time to discuss with colleagues to improve the quality of my educational robotics activities.	0.783			0.277
**RCom05**—I am willing to explore the benefits of educational robotics for learning and teaching.	0.653			0.256
**REff01**—I possess sufficient knowledge to apply programming in robotics.		0.997		0.228
**REff04**—Implementing educational robotics in my teaching process would be easy for me.		0.886		0.168
**REff03**—I am confident in implementing educational robotics in the classroom.		0.789		0.221
**REff02**—I understand the pedagogical benefit of different types of robots.		0.749		0.312
**REff05**—I could assess learning outcomes in robotics learning activities.		0.630		0.277
**RAff02**—I am excited about the implementation of educational robotics activities in the classroom.			0.786	0.137
**RAff01**—I would enjoy implementing robotics educational activities in the classroom.			0.673	0.181
**RAff03**—I would be happy with the implementation of educational robotics activities.			0.644	0.166
**RAff04**—I am optimistic about the implementation of educational robotics activities.			0.569	0.344
	**RCOM** (m = 3.66, SD = 1.02)	**RSE** (m = 2.96, SD = 1.13)	**RAC** (m = 3.48, SD = 1.11)	

Note. Applied rotation method is promax. RCOM = commitment, RSE = self-efficacy, RAC = affective conditions.

**Table 3:** Factor correlation matrix.

	RCOM	RSE	RAC
RCOM	1.000		
RSE	0.670 ***	1.000	
RAC	0.835 ***	0.772 ***	1.000

Note: RCOM = commitment, RSE = self-efficacy, RAC = affective conditions, *** *p* < 0.001.

**Table 4:** CFA measurement model: factors, estimates of factor loadings, standards errors, lower and upper 95% CI, and statistical significance.

Factor Loadings								
						95% Confidence Interval		
Factor	Indicator	Estimate	Std. Error	z-Value	*p*	Lower	Upper	Std. Est. (All)
Commitment	RCom01	0.889	0.044	20.026	<0.001	0.802	0.976	0.823
	RCom02	0.955	0.043	21.965	<0.001	0.870	1.040	0.872
	RCom03	0.919	0.050	18.512	<0.001	0.821	1.016	0.781
	RCom04	0.975	0.045	21.626	<0.001	0.886	1.063	0.864
	RCom05	0.976	0.046	21.133	<0.001	0.886	1.067	0.852
Self-Efficacy	REff01	1.146	0.054	21.193	<0.001	1.040	1.252	0.852
	REff02	1.058	0.052	20.496	<0.001	0.956	1.159	0.834
	REff03	1.060	0.048	22.006	<0.001	0.965	1.154	0.872
	REff04	1.199	0.049	24.489	<0.001	1.103	1.295	0.928
	REff05	0.981	0.048	20.594	<0.001	0.888	1.075	0.837
Affective Conditions	RAff01	1.102	0.046	23.725	<0.001	1.011	1.193	0.909
	RAff03	1.103	0.045	24.282	<0.001	1.014	1.192	0.921
	RAff04	0.913	0.047	19.331	<0.001	0.820	1.005	0.801
	RAff02	1.164	0.047	24.654	<0.001	1.071	1.256	0.929

**Table 5:** Measurement invariance for gender.

Invariance Model	*χ* ^2^	*df*	CFI	TLT	RMSEA	SRMR	Δ*χ*^2^	Δ*df*	*p*-Value
	0	0							
Configural	600,131	148	0.961	0.953	0.086	0.040	600,131	148	
Metric	605,209	159	0.962	0.956	0.083	0.043	5078	11	0.90
Scalar	628,302	170	0.961	0.958	0.081	0.044	23,093	11	<0.05
Strict	671,234	184	0.958	0.959	0.081	0.045	42,932	14	<0.001

## Data Availability

The data presented in this study are available upon request from the corresponding author.

## Supplementary Materials

The following supporting information can be downloaded at https://www.mdpi.com/article/10.3390/bs15091227/s1, Figure S1. Whole Sample. Bootstrapped CIs of estimated edge-weights for the network. The red line corresponds to the sample values, and the black line and the grey area correspond to the mean and the bootstrapped CIs. Each horizontal line represents one edge of the network ordered by edge-weights. Figure S2. Network of the whole Sample: Bootstrapped difference tests on the nodal strength of all the variables in the network. The Black boxes correspond to nodes that differed significantly from another node in the matrix. Ther numbers in white boxes in the plot indicate the strength of the corresponding node. Figure S3. Edge stability|: Average correlations between networks estimated with sampled cases and original sample. Red line specifies the means and area indicating the range from the 2.5th to the 97.5th percentile. Figure S4. Male participants: Bootstrapped CIs of estimated edge-weights for the network. The red line corresponds to the sample values, and the black line and the grey area correspond to the mean and the bootstrapped CIs. Each horizontal line represents one edge of the network ordered by edge-weights. Figure S5. Network of the male participants: Bootstrapped difference tests on the nodal strength of all the variables in the network. The Black boxes correspond to nodes that differed significantly from another node in the matrix. Ther numbers in white boxes in the plot indicate the strength of the corresponding node. Figure S6. Male network Edge stability|: Average correlations between networks estimated with sampled cases and original sample. Red line specifies the means and area indicating the range from the 2.5th to the 97.5th percentile. Figure S7. Female participants: Bootstrapped CIs of estimated edge-weights for the network. The red line corresponds to the sample values, and the black line and the grey area correspond to the mean and the bootstrapped CIs. Each horizontal line represents one edge of the network ordered by edge-weights. Figure S8. Network of the female participants: Bootstrapped difference tests on the nodal strength of all the variables in the network. The Black boxes correspond to nodes that differed significantly from another node in the matrix. Ther numbers in white boxes in the plot indicate the strength of the corresponding node. Figure S9. Female network Edge stability|: Average correlations between networks estimated with sampled cases and original sample. Red line specifies the means and area indicating the range from the 2.5th to the 97.5th percentile.
