# Supplementary material for: Teachers’ Readiness to Implement Robotics in Education: Validation and Measurement Invariance of TRi-Robotics Scale via Confirmatory Factor Analysis and Network Psychometrics
Source: Behav Sci (Basel). 2025 Sep 10;15(9):1227. doi: 10.3390/bs15091227 (PMC12466464; doi:10.3390/bs15091227)
Supplement: Supplementary file 1 [file behavsci-15-01227-s001.zip › behavsci-3775318-supplementary.pdf]

Supplementary Material

Analysis for network stability and accuracy for TRi-Robotics scale and the whole sample

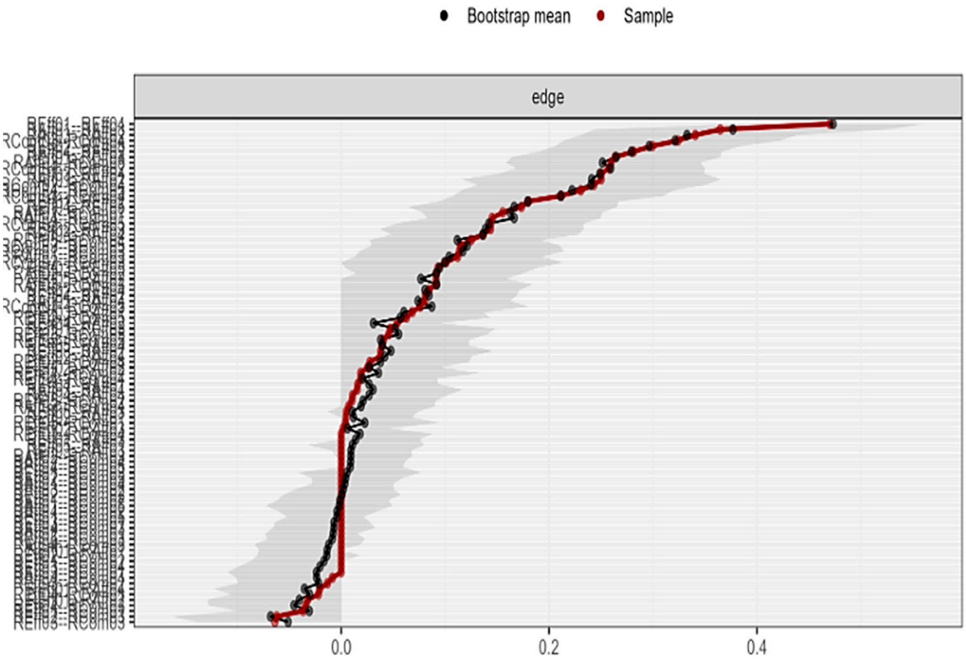

Figure S1. Whole Sample. Bootstrapped CIs of estimated edge-weights for the network. The red line corresponds to the sample values, and the black line and the grey area correspond to the mean and the bootstrapped CIs. Each horizontal line represents one edge of the network ordered by edge-weights.

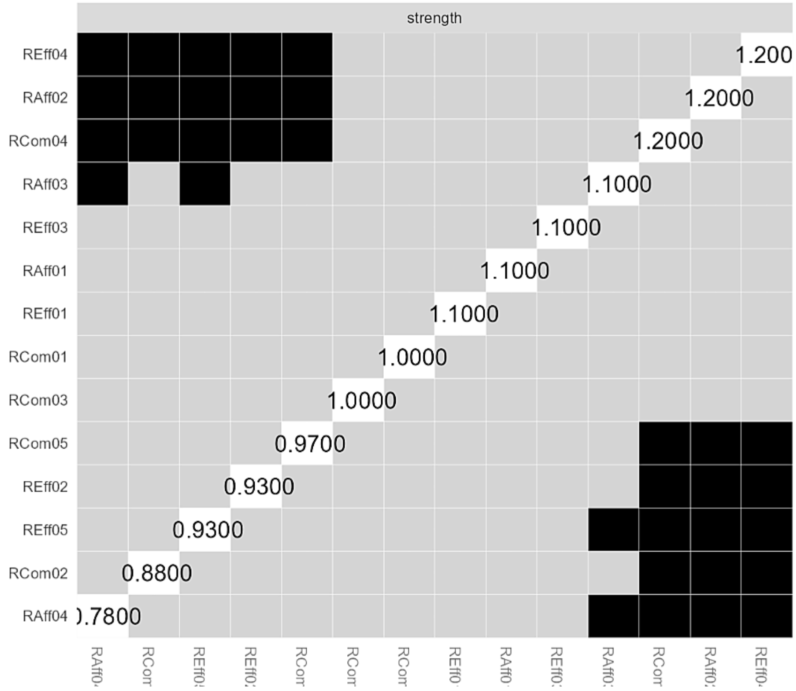

Figure S2. Network of the whole Sample: Bootstrapped difference tests on the nodal strength of all the variables in the network. The Black boxes correspond to nodes that differed significantly from another node in the matrix. Ther numbers in white boxes in the plot indicate the strength of the corresponding node.

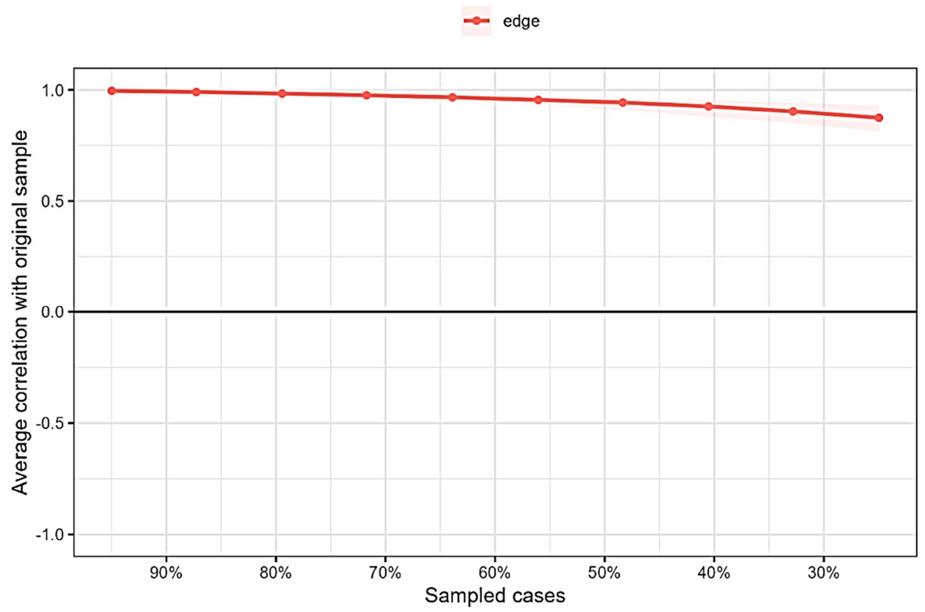

Figure S3. Edge stability|: Average correlations between networks estimated with sampled cases and original sample. Red line specifies the means and area indicating the range from the 2.5th to the 97.5th percentile.

The results for network stability and accuracy for male participants

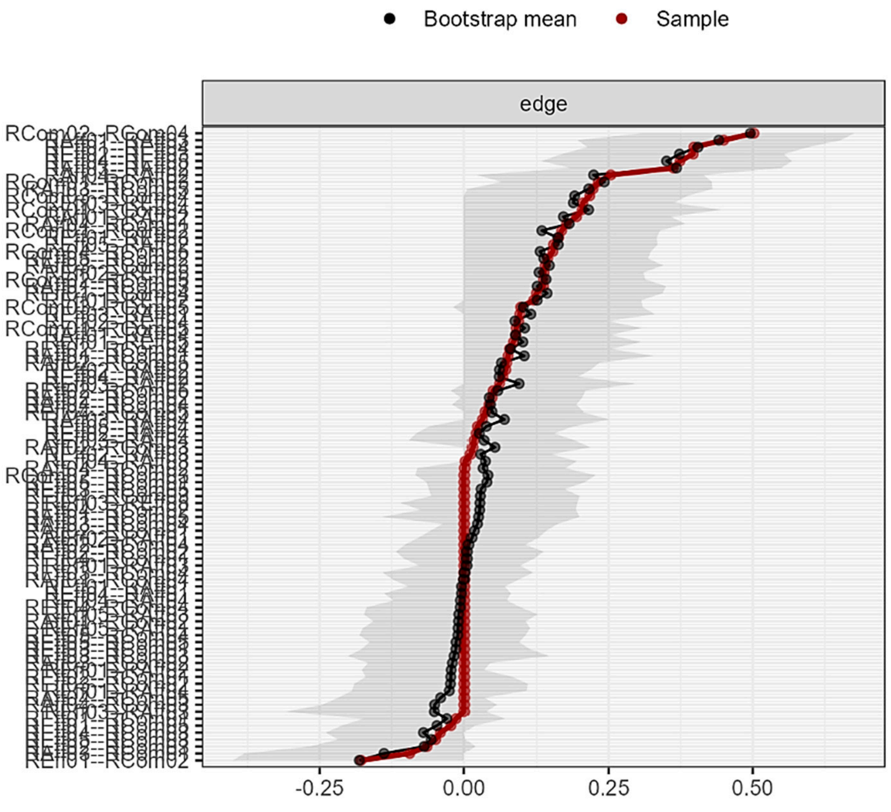

Figure S4. Male participants: Bootstrapped CIs of estimated edge-weights for the network. The red line corresponds to the sample values, and the black line and the grey area correspond to the mean and the bootstrapped CIs. Each horizontal line represents one edge of the network ordered by edge-weights.

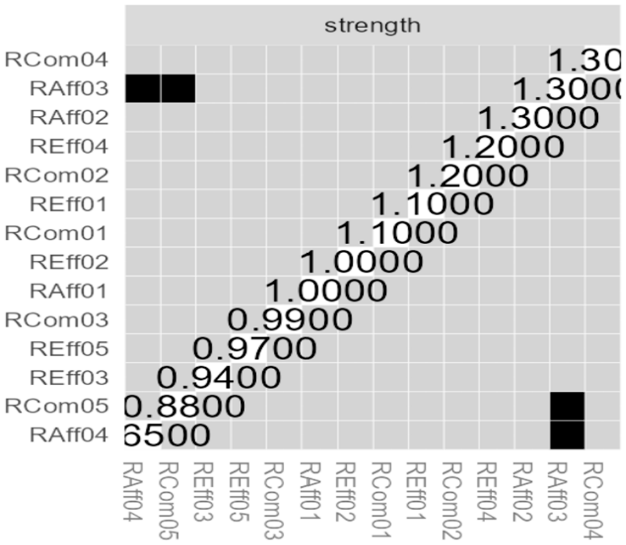

Figure S5. Network of the male participants: Bootstrapped difference tests on the nodal strength of all the variables in the network. The Black boxes correspond to nodes that differed significantly from another node in the matrix. Ther numbers in white boxes in the plot indicate the strength of the corresponding node.

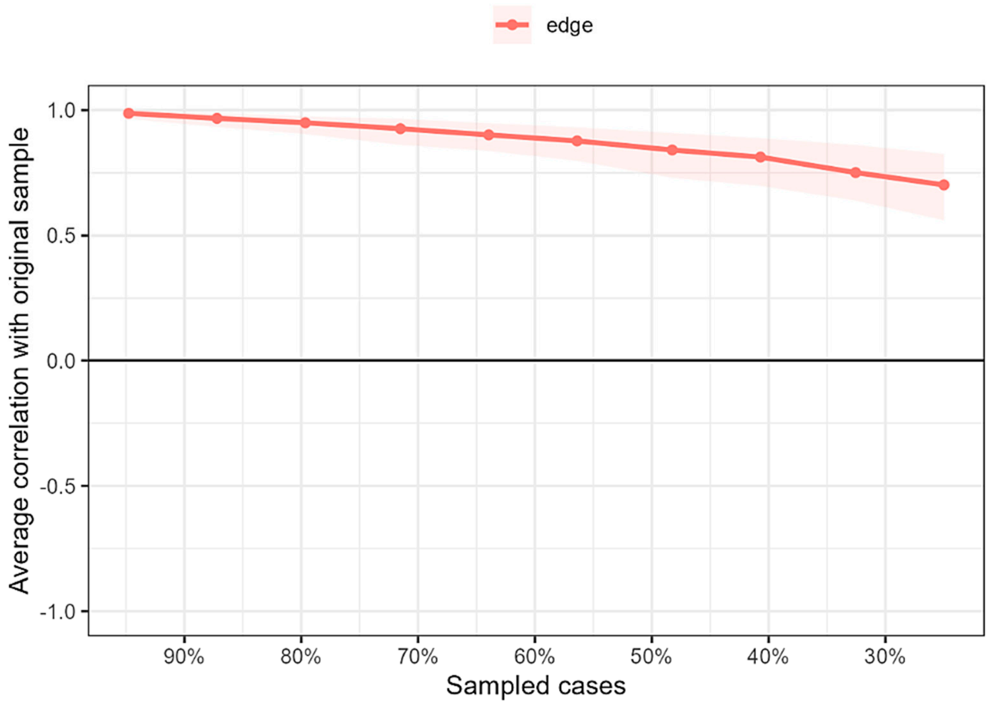

Figure S6. Male network Edge stability|: Average correlations between networks estimated with sampled cases and original sample. Red line specifies the means and area indicating the range from the 2.5th to the 97.5th percentile.

The results for network stability and accuracy for female participants

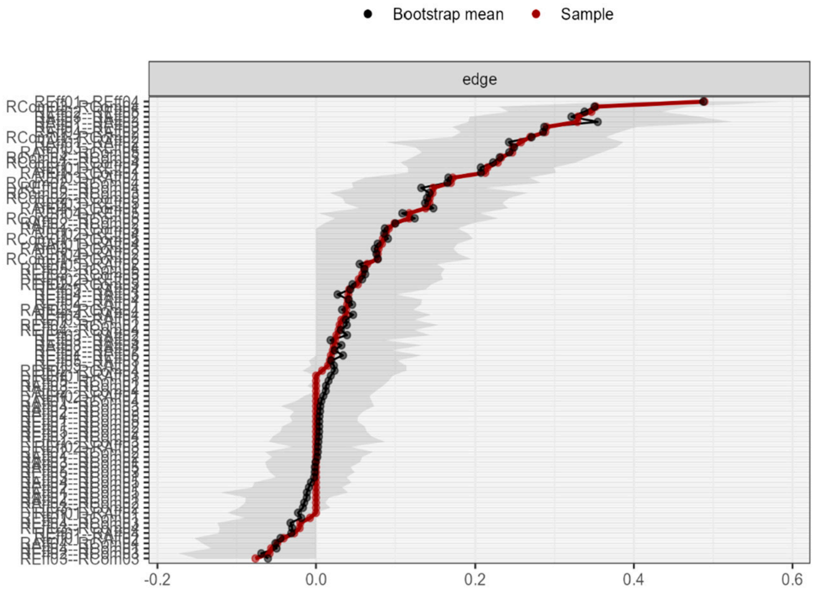

Figure S7. Female participants: Bootstrapped CIs of estimated edge-weights for the network. The red line corresponds to the sample values, and the black line and the grey area correspond to the mean and the bootstrapped CIs. Each horizontal line represents one edge of the network ordered by edge-weights.

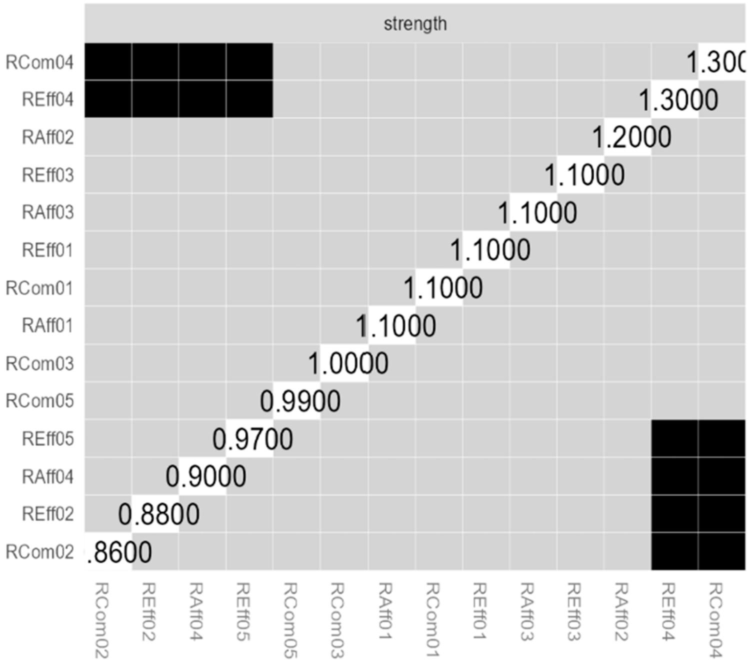

Figure S8. Network of the female participants: Bootstrapped difference tests on the nodal strength of all the variables in the network. The Black boxes correspond to nodes that differed significantly from another node in the matrix. Ther numbers in white boxes in the plot indicate the strength of the corresponding node.

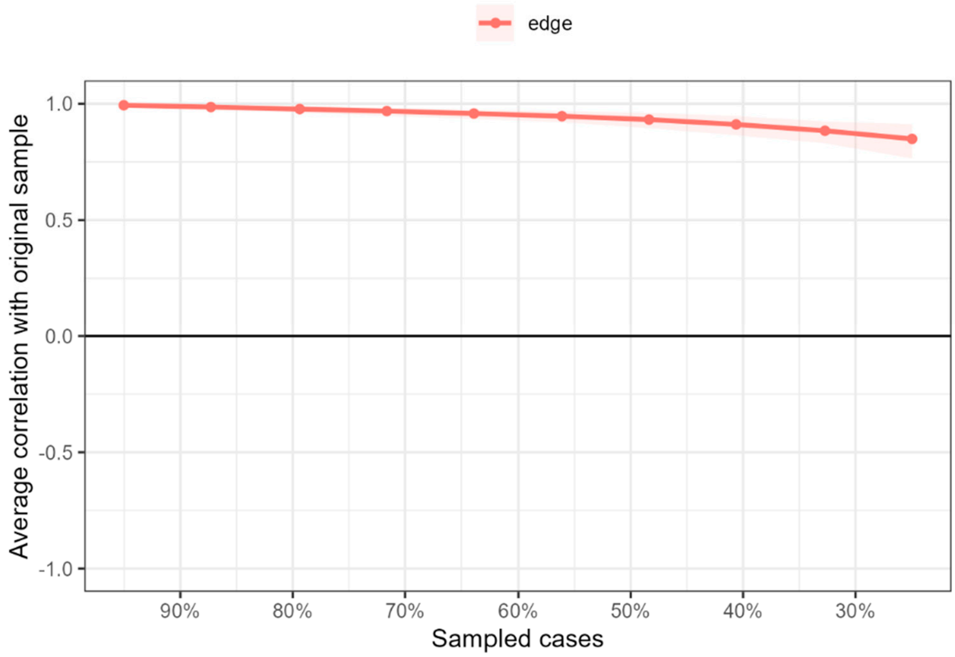

Figure S9. Female network Edge stability|: Average correlations between networks estimated with sampled cases and original sample. Red line specifies the means and area indicating the range from the 2.5th to the 97.5th percentile.
